# Clinical efficacy study of the lateral parapatellar approach combined with the “Hedgehog” technique in treating complex comminuted patellar fractures

**DOI:** 10.3389/fsurg.2025.1680334

**Published:** 2025-11-03

**Authors:** Teng Ma, Lili Chao, Daihao Wei

**Affiliations:** 1 Department of Orthopedic Surgery, General Hospital of Ningxia Medical University, Yinchuan, Ningxia, China; 2 Department of Anesthesia and Perioperative Medicine, General Hospital of Ningxia Medical University, Yinchuan, Ningxia, China; 3 The First School of Clinical Medicine, General Hospital of Ningxia Medical University, Yinchuan, Ningxia, China

**Keywords:** lateral parapatellar approach, “Hedgehog” technique, patellar fracture, clinical efficacy, treating

## Abstract

**Objective:**

The present study aimed to investigate the efficacy of the lateral parapatellar approach combined with multiple Kirschner wire tension bands (“Hedgehog” technique) in the treatment of complex comminuted patellar fractures, improving surgical outcomes, and promoting early recovery of knee joint function.

**Methods:**

This study adopted a retrospective design. Based on the inclusion and exclusion criteria, patients with complex comminuted patellar fractures admitted to the hospital from January 2018 to January 2024 were enrolled. Among them, 21 patients in Group A were treated via the lateral parapatellar approach combined with multiple Kirschner wire tension bands and Ethibond suture fixation, whereas 24 patients in Group B underwent conventional midline anterior patellar approach combined with Kirschner wire tension band fixation. The two groups were compared and analyzed in terms of incision length, intraoperative blood loss, operation time, reduction quality, VAS score, postoperative complications, Böstman score, initiation time of postoperative knee functional exercises and the last follow-up KOOS score.

**Results:**

All patients had complete follow-up data, with a mean follow-up duration of 18 months, and there were no statistically significant differences in baseline characteristics between the two groups. Compared with Group B, Group A had significantly better outcomes in terms of operation time, reduction quality, VAS score (3 days postoperatively), incidence of postoperative complications (scarring), Böstman score (3, 6, and 12 months postoperatively), fracture healing time, initiation time of knee functional exercises and KOOS score (*P* < 0.05). No statistically significant differences were observed between the two groups in terms of incision length, intraoperative blood loss, VAS score (1 day postoperatively), or postoperative complications (pin migration, irritation, and infection) (*P* > 0.05).

**Conclusion:**

In the surgical management of complex comminuted patellar fractures, the lateral parapatellar approach allows for precise reduction of fracture fragments under direct visualization, achieving anatomical reduction with reliable fixation. This approach facilitates early functional exercises, accelerates rehabilitation, and achieves favorable clinical outcomes while avoiding patellectomy, and thus may represent a valuable alternative technique. The simple and cost-effective internal fixation technique using a Kirschner wire tension band combined with Ethibond suture remains vigorously viable in the era of rapidly advancing orthopaedic treatment technologies.

## 1 Introduction

Patellar fracture is a common clinical fracture with diverse morphologies, mostly caused by direct or indirect violence, accounting for approximately 1%–1.65% of all adult fractures (1). With the development of the social construction and transportation industries, the incidence of complex comminuted patellar fractures has shown an increasing trend, and open reduction and internal fixation is the preferred surgical treatments (2). Complex comminuted patellar fractures are characterized by multiple fracture lines, large separation gaps between fracture fragments, poor fracture stability, tearing of the surrounding medial and lateral retinacula, soft tissue edema, and impaired blood supply. These severely damage the extensor mechanism centered on the patella, and postoperative complications such as nonunion or malunion, joint stiffness, post-traumatic arthritis, and knee pain are prone to occur. Some patients even require patellectomy or joint replacement (3–5). The keys to treatment lie in achieving precise anatomical reduction, promoting patellar stability, minimizing secondary damage to surrounding soft tissues, and enabling early functional exercises. Therefore, the choice of surgical approach, selection of internal fixation, and reliability of fracture fixation determine the clinical therapeutic effect.

Common surgical approaches for patellar fractures include midline anterior longitudinal incision, transverse incision, and medial parapatellar approach (6). Continuously updated internal fixation materials and techniques have been introduced into clinical practice (7–10). However, these methods have limitations in terms of universality. As a classic internal fixation method, the Kirschner wire tension band technique still achieves strong internal fixation.

Currently, in the treatment of complex comminuted patellar fractures, previous researchers have continuously improved surgical approaches and internal fixation options; however, the outcomes remain suboptimal. Postoperatively, patients exhibit only moderate knee joint function, and some still require joint replacement or patellectomy several years after surgery. Therefore, there is no consensus on the treatment strategy for this type of fracture (11–13). The lateral parapatellar approach for complex comminuted patellar fractures allows adequate exposure of the patellar surface of the patellofemoral joint, facilitating reduction and fixation under direct visualization. It minimizes secondary damage to the extensor mechanism and peripatellar soft tissues, avoids patellectomy, and enables patients to perform early knee functional exercises. This study aimed to evaluate the efficacy and safety of the lateral patellar approach combined with multiple Kirschner wire tension bands (“Hedgehog” technique) and Ethibond suture fixation in the treatment of complex comminuted patellar fractures.

## 2 Patients and methods

### 2.1 Clinical characteristics

This study was a retrospective study, approved by the Ethics Committee of the General Hospital of Ningxia Medical University (Ethics No.: KYLL-2025-1659). A total of 45 patients with complex comminuted patellar fractures admitted to the General Hospital of Ningxia Medical University from January 2018 to January 2024 were enrolled and divided into Groups A and B. Twenty-one patients in Group A were treated via the lateral patellar approach combined with multiple Kirschner wire tension band and Ethibond suture fixation, whereas 24 patients in Group B received the conventional midline anterior patellar approach combined with Kirschner wire tension band fixation. There were no statistically significant differences in the general data between the two groups (*P* > 0.05), as shown in Supplementary Table 1. All patients had good compliance and completed comprehensive follow-up, with internal fixation removal performed at the end of the follow-up period.

### 2.2 Inclusion criteria

① No other fractures in the ipsilateral limb or fractures in the contralateral limb; ② comminuted patellar fractures with an AO classification (14) of C3.2 or higher; ③ all patients underwent fixation with multiple Kirschner wire tension bands and Ethibond sutures; ④ no history of knee joint-related diseases before trauma. All surgeries were performed by the same team.

### 2.3 Exclusion criteria

① Complicated with multiple limb fractures; ② open fractures; ③ polytrauma; ④ previous knee surgery; ⑤ previous knee deformity or joint disease that affected normal activities; ⑥ presence of anterior or posterior cruciate ligament injury of the knee; ⑦ patients with limb hemiplegia due to cerebral infarction.

### 2.4 Perioperative management

**Figure 1:**
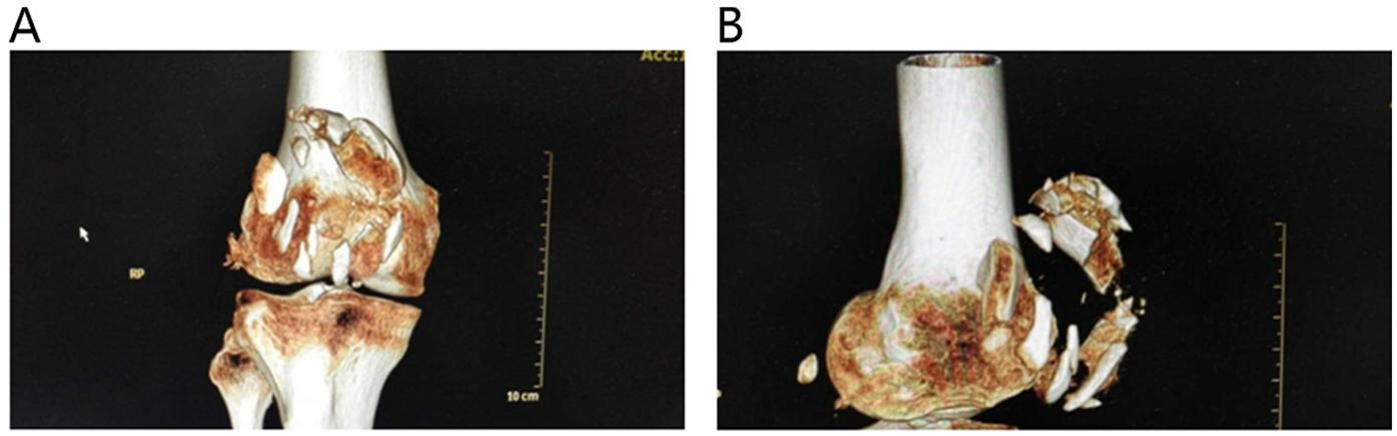
Preoperative three-dimensional reconstructed CT images of patellar fractures.

Upon admission, patients received local cold compresses on the affected limb, along with extension-position immobilization via a brace and elevation of the affected limb to reduce swelling. When necessary, oral or intravenous detumescent drugs were administered. Subcutaneous injection of low-molecular-weight heparin calcium was given for thrombosis prophylaxis. Analgesic treatment was provided on the basis of the degree of pain. Preoperatively, anteroposterior and lateral x-rays of the knee joint, as well as three-dimensional CT reconstruction of the knee joint, were completed to assess the fracture morphology (Figure 1), and preoperative surgical planning was conducted. Knee joint MRI was completed to evaluate ligament injury. Cefazolin sodium was administered 30 min before surgery for infection prophylaxis. Postoperatively, symptomatic treatments such as detumescence were continued, and the incision was inspected for signs of redness, swelling, or infection during dressing changes on the second postoperative day. Patients were instructed on methods of knee joint functional exercises after discharge and were informed of the postoperative follow-up schedule and precautions.

### 2.5 Surgical technique

All patients were placed in the supine position after receiving spinal anesthesia or general anesthesia, and a pneumatic tourniquet was applied to the proximal femur.

#### 2.5.1 Lateral parapatellar approach

**Figure 2:**
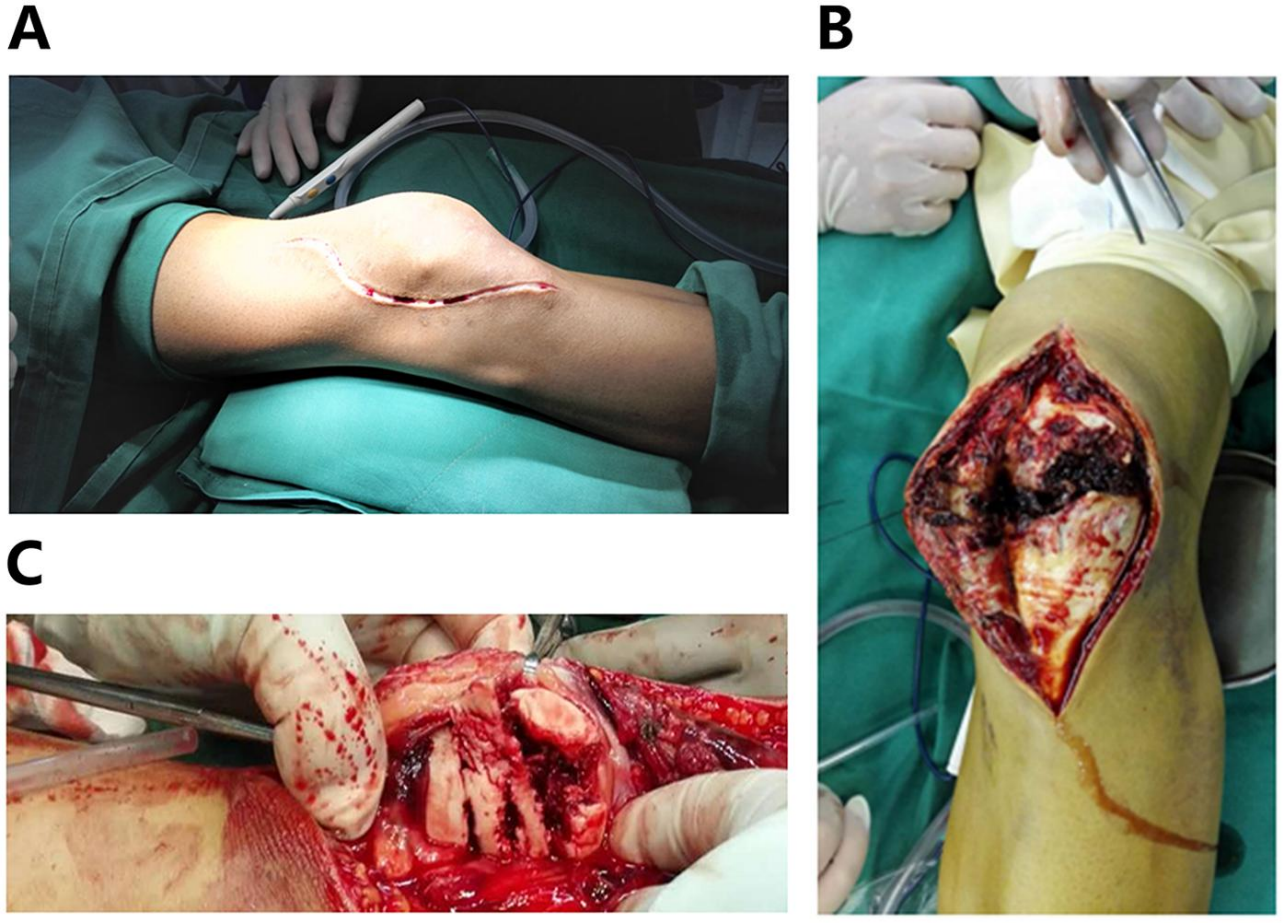
**(A)** Lateral parapatellar incision. **(B)** Reflection of the lateral skin flap. **(C)** Exposure of the patellar articular surface.

**Figure 3:**
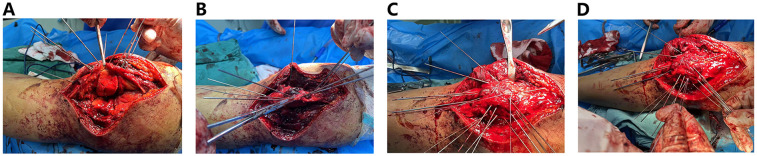
**(A)** Kirschner wires are fixed above the ligament. **(B,C)** “Hedgehog-like” fixation structure. **(D)** Ethibond suture-assisted fixation.

**Figure 4:**
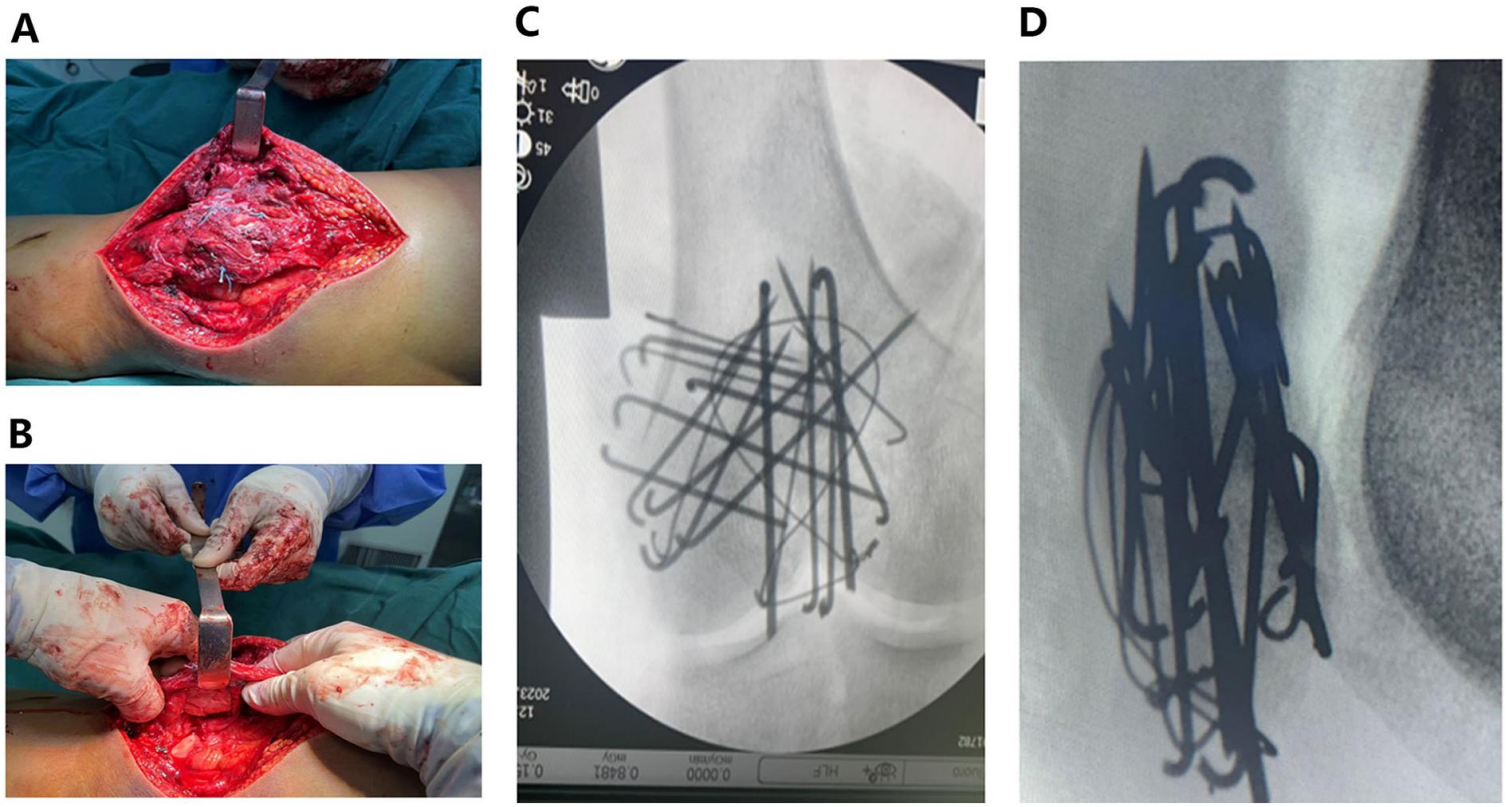
**(A)** The periosteum on the surface remains intact. **(B)** The articular surface is anatomically reduced. **(C,D)** Anteroposterior and lateral x-ray fluoroscopy images indicating that internal fixation is appropriate and that the degree of reduction is satisfactory.

**Figure 5:**
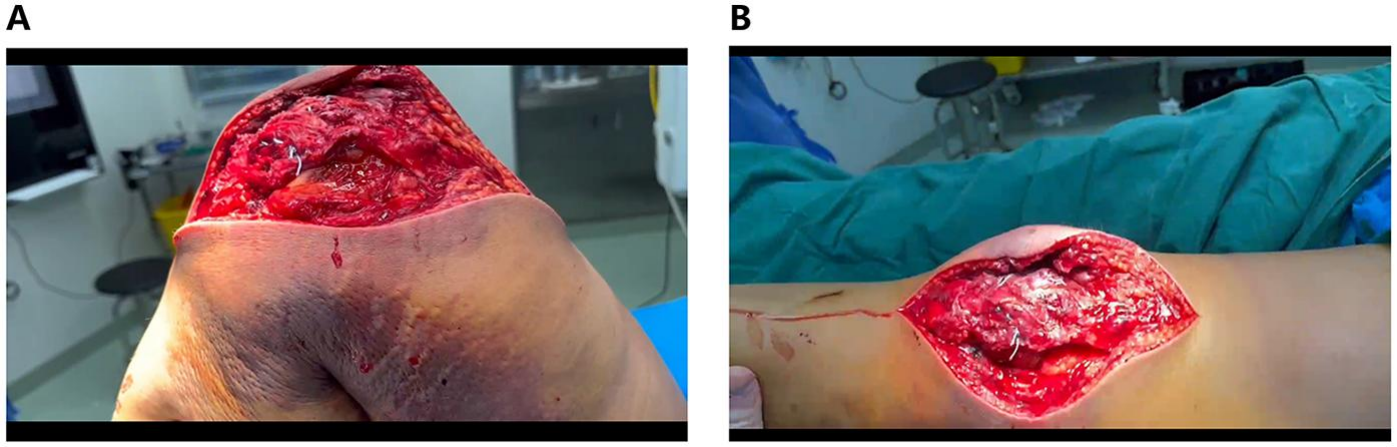
**(A,B)** The complete knee flexion and extension function exercises were immediately performed, indicating that the internal fixation was firm and stable.

A lateral parapatellar incision was made (Figure 2A), avoiding the anterior area with poor skin conditions. A full-thickness incision of the skin and subcutaneous tissue was made, followed by reflection of the lateral skin flap (Figure 2B). The lateral retinaculum was incised longitudinally. Blood clots and hemarthrosis in the fracture ends and joint cavity were thoroughly debrided. The patellar articular surface was reflected (Figure 2C) to expose the fracture. Fracture fragments were reduced sequentially under direct vision to achieve articular surface congruence. On the basis of the size of the fragments, multiple Kirschner wires (1.0 mm, 1.5 mm, and 2.0 mm in diameter) were selected and inserted through the superior surface of the lateral retinaculum for fixation (Figure 3A), forming a “hedgehog-like” fixation structure (Figures 3B,C). Two 2.0-mm “main” Kirschner wires were placed, and a steel wire tension band was used to bundle and fix the layered superficial comminuted fragments. Ethibond No. 5 suture was applied for figure-of-eight and circular reinforcement fixation (Figure 3D) to evolve the “hedgehog-like” structure. The redundant ends of the Kirschner wires were bent and trimmed, revealing the Ethibond sutures with figure-of-eight and circular fixation. The periosteum on the surface was fully covered (Figure 4A), providing a strong blood supply foundation for bone healing. Reflection of the patella confirmed an intact articular surface with anatomical reduction (Figure 4B). Intraoperative fluoroscopy was performed to verify the reduction (Figures 4C,D). Immediate full-range flexion and extension of the knee joint were conducted intraoperatively to assess fixation stability (Figure 5). After irrigation and hemostasis, the lateral retinaculum was sutured, the joint cavity was closed, and the subcutaneous tissue and skin were sutured layer by layer.

#### 2.5.2 Midline anterior longitudinal approach

After the skin condition had improved sufficiently (For these patients, we gave them a longer preoperative waiting time of about 7–10 days to avoid increasing the chance of surgical infection), a midline anterior incision was made. The skin and subcutaneous tissues were incised full-thicknessly. The patellar fracture fragments were exposed via blunt dissection from the superior surface. The lateral retinaculum was incised, and blood clots and hematomas within the fracture site and joint cavity were thoroughly debrided. The size and displacement of the fracture fragments were palpated manually. Reduction forceps were applied to grasp and stabilize the fragments from the superior surface. Kirschner wires of varying diameter were inserted at multiple angles for provisional fixation. An 8-mm steel wire was used for figure-of-eight tension band fixation. Intraoperative fluoroscopy was performed to confirm satisfactory reduction. After irrigation and hemostasis, the lateral retinaculum was repaired, the joint cavity was closed, and the subcutaneous tissues and skin were sutured in layers.

### 2.6 Evaluation indicators

- Intraoperative parameters: Incision length, intraoperative blood loss, and operative duration were recorded for both groups.
- Radiological assessment: Postoperative x-ray (anteroposterior and lateral views) and knee CT reconstructions (axial and coronal planes) were performed to evaluate fracture reduction quality and internal fixation integrity.
- Pain assessment: The visual analog scale (VAS) (15) was used to score pain intensity at 1 and 3 days postoperatively.
- Knee function assessment: The Böstman Knee Scoring Scale (16) was used to evaluate knee function at 3, 6, and 12 months postoperatively.
- Functional recovery: The time to achieve 90° knee flexion during early rehabilitation was recorded.
- Fracture healing: Union time was determined radiographically and compared between groups.
- Scar evaluation: The Vancouver scar scale (VSS) (17) was used to assess incision scarring after the patient resumed full weight-bearing activities. Hypertrophic scarring not only impairs appearance but also affects joint functional exercise, and even compromises patients’ quality of life (18, 19).
- Quality of life: A comprehensive assessment using the Knee Injury and Osteoarthritis Outcome Score (KOOS) (20) was performed at the final follow-up. This scale consists of 42 items, with each item scored on a 5-point Likert scale (4, 3, 2, 1, 0), yielding a total possible raw score of 168. It comprises 5 subscales: pain, symptoms, activities of daily living, sports and recreation function, and quality of life. Each subscale is calculated independently via the following formula: transformed score = 100 − (actual raw score × 100/possible raw score range). A higher score indicates better knee joint function and quality of life.

### 2.7 Postoperative functional exercise

In group A, passive knee flexion-extension exercises (0–150°) were performed immediately during the operation. Depending on the recovery situation, patients in this group were allowed to perform partial weight—bearing activities with crutches 2 weeks after the operation, and meanwhile, passive knee flexion-extension exercises (0–90°, shown in the early stage) were carried out. At 4 weeks after the operation, full weight-bearing activities were permitted, along with knee flexion—extension exercises (0–140°). Then, normal knee function exercises and normal walking movements were gradually performed.

Approximately 1.5 years after the operation, internal fixation removal was carried out for all patients in this group according to their own requirements.

In group B, the knee joint was immobilized in the extended position for 2–4 weeks after the operation. On the basis of the recovery condition, patients in this group were allowed to perform partial weight-bearing activities with crutches 4 weeks post-operation, and passive knee flexion-extension exercises (0–90°) were conducted simultaneously. At 8 weeks after the operation, full weight—bearing activities were permitted, together with knee flexion—extension exercises (0–130°). Then, moderate squatting activities with the support of a table were started. Approximately 3 months after the operation, patients gradually stopped squatting with the support of the table. Approximately 4 months after the operation, full squatting activities were performed according to the patients' pain tolerance and fracture healing conditions.

Approximately 1.5 years after the operation, internal fixation removal was carried out for all patients in this group according to their own requirements.

### 2.8 Statistical methods

In this study, data analysis was performed via the statistical software SPSS 26.0 to ensure the scientific rationality and credibility of the statistical results. Categorical variables are presented as percentages, and the chi-square test was used to compare differences between groups. For continuous variables, all data are expressed as the mean ± standard deviation. For continuous variables that conformed to a normal distribution, the independent-samples *t*-test was employed; in cases where the data deviated from a normal distribution, the Mann–Whitney *U* test was adopted instead. Across all the statistical analyses, a *P*-value below 0.05 was regarded as indicating statistical significance.

## 3 Results

There were no statistically significant differences in the baseline characteristics between the two groups. Internal fixation removal was performed for all patients at approximately 1.5 years.

### 3.1 Analysis of surgical data

The operative time in Group A was significantly shorter than that in Group B (*P* < 0.05). No statistically significant differences in incision length or intraoperative blood loss were detected between the two groups; however, the blood loss in Group B was numerically lower (Table 1).

**Table 1:** Analysis of surgical data.

Indicator	Group A	Group B	*P*
Incision length (cm)	12.24 ± 2.36	11.96 ± 2.27	0.688
Blood loss (ml)	155.71 ± 54.36	131.46 ± 36.04	0.081
operative time (min)	74.90 ± 13.03	89.17 ± 14.73	0.001
Reduction degree (<1 mm/>1 mm)	19/2	15/9	0.040

### 3.2 Postoperative VAS scores

There was no significant difference in the VAS score between the two groups on the first postoperative day (*P* > 0.05), whereas a statistically significant difference was noted on the third postoperative day (*P* < 0.001) (Table 2). Postoperative pain alleviated gradually over time. Oral analgesics were administered on the day of surgery, and no injectable analgesics were needed.

**Table 2:** Data of postoperative follow-up results.

Indicator	Group A	Group B	*P*
VAS score			
1st day postoperatively	1.24 ± 0.43	1.21 ± 0.41	0.816
3rd day postoperatively	2.33 ± 0.48	3.42 ± 0.83	<0.001
Fracture healing time (days)	118.14 ± 9.96	126.88 ± 11.15	0.009
Böstman score			
3 months postoperatively	22.00 ± 1.61	20.25 ± 1.11	<0.001
6 months postoperatively	25.38 ± 2.41	23.08 ± 0.92	<0.001
12 months postoperatively	27.19 ± 1.03	25.67 ± 1.34	<0.001
Time to achieve 0–90° knee flexion and extension (days)	14.24 ± 1.261	32.33 ± 4.67	<0.001

### 3.3 Evaluation of fracture reduction and healing via x-ray and Ct

- Immediate postoperative assessment: x-ray images confirmed satisfactory fracture reduction. Lateral views revealed a smooth patellar articular surface of the patellofemoral joint without protrusion, and the length of Kirschner wire tails was appropriate.
- CT assessment on postoperative day 2: Three-dimensional CT reconstruction revealed that the displacement between articular fracture fragments was <2 mm in all patients. Group A achieved better fracture reduction, with a statistically significant difference (*P* < 0.05) (Table 1). • Fracture healing time: Fracture healing was defined as the disappearance of fracture lines. Serial x-ray evaluations were performed at 1, 2, 3, 6, and 12 months postoperatively, with no redisplacement observed between bone fragments. The mean healing time was 118.14 ± 9.96 days in Group A and 126.88 ± 11.15 days in Group B, which was a statistically significant difference (*P* < 0.05) (Table 2), indicating faster healing in Group A.

### 3.4 Böstman scores and KOOS scores

- Böstman scores: The scores of the two groups at 3, 6, and 12 months postoperatively were significantly different. Group A achieved better scores, with better and faster recovery of knee joint function (Table 2, *P* < 0.001).
- KOOS scores: At the final follow-up, Group A showed better recovery of knee joint function and quality of life, with statistically significant differences in the scores of all 5 subscales of the KOOS between the two groups (Table 3, *P* < 0.001).

All patients were satisfied with the treatment outcomes and postoperative functional recovery.

**Table 3:** KOOS score of final follow-up.

Indicator	Group A	Group B	*P*
Pain	96.42 ± 1.28	81.82 ± 9.58	<0.001
Symptoms	98.47 ± 1.81	72.32 ± 7.31	<0.001
Activities of daily living	96.84 ± 1.34	79.47 ± 5.68	<0.001
Sport and recreation function	89.29 ± 4.55	72.08 ± 5.30	<0.001
Knee—related quality of life	90.18 ± 4.23	71.09 ± 9.18	<0.001

### 3.5 Postoperative complications

The Vancouver scar scale (VSS) demonstrated more prominent incision scar hyperplasia (height, pliability) in Group B than in Group A, with a statistically significant difference (Table 4, *P* < 0.001). No incision infections occurred in any patient. In Group A, one patient experienced wire breakage at 6 months, which did not affect daily life. In Group B, one patient experienced marginal skin darkening on postoperative day 2 (considered superficial necrosis), with satisfactory wound healing after 3 weeks of dressing changes; two patients reported skin irritation, which slightly restricted knee flexion-extension. At the final follow-up, after internal fixation removal, the range of motion of the knee was not significantly restricted.

**Table 4:** Results of Vancouver scar scale scores for postoperative incision healing in the two groups.

Indicator	Group A	Group B	*P*
Pigmentation	1.76 ± 0.63	1.63 ± 0.50	0.417
Height	1.10 ± 0.30	1.75 ± 0.44	<0.001
Vascularity	2.19 ± 0.75	2.33 ± 0.64	0.493
Pliability	1.52 ± 0.68	2.38 ± 0.71	<0.001

## 4 Discussion

As the largest sesamoid bone in the human body, the patella plays a crucial role in the extensor mechanism of the knee. It is proximally connected to the quadriceps tendon and distally attached to the patellar ligament, with its insertion at the tibial tuberosity. The trochlear function of the patella extends the moment arm of the quadriceps, enhancing knee extension strength by 30%–50%. Therefore, the stability of the patella and the integrity of its surrounding ligaments are prerequisites for the proper function of the extensor mechanism (14, 21).

Historically, surgical management of complex comminuted patellar fractures has yielded suboptimal outcomes, often resulting in poor knee function that significantly impairs patient health. Some patients undergo partial or total patellectomy. Such fractures remain challenging to treat, with a variety of surgical approaches employed; outcomes are closely associated with hospital tier and surgeon expertise. The keys to managing these injuries lies in achieving anatomical reduction, ensuring stable internal fixation, minimizing secondary damage to peri-fracture soft tissues and neurovascular structures, promoting fracture healing, reducing iatrogenic injury to the extensor mechanism, and facilitating joint function recovery. The therapeutic approach described herein—utilizing a lateral parapatellar approach combined with multi-Kirschner wire tension band (“hedgehog” technique) and Ethibond suture fixation—has demonstrated favorable surgical outcomes, obviating the need for patellectomy and thus merits clinical promotion and application.

Currently, surgical approaches for patellar fractures include the anterior midline longitudinal incision, anterior transverse incision, medial parapatellar approach, and lateral parapatellar approach. Among these, the anterior midline approach is the most commonly used, with numerous reports documenting its favorable outcomes; however, there is a paucity of literature on its application in treating complex comminuted patellar fractures. This approach does not allow for direct visualization of fracture reduction; instead, it relies on the surgeon's tactile perception of the articular surface, making it suitable for non-comminuted, large-fragment fractures. Additionally, x-ray evaluation at specific positions is needed to assess fracture reduction (22–24). The transverse incision has also been widely reported, but its indications are relatively narrow and associated with greater trauma, leading to its gradual replacement by longitudinal incisions.

In the present study, the lateral parapatellar approach offered advantages for surgical manipulation because of its proximity to the operator. Moreover, after patellar eversion, fractures can be precisely reduced under direct visualization, enabling anatomical reduction of the patellar articular surface. As evidenced by the postoperative CT reconstruction data, the quality of fracture reduction in Group A was superior to that in Group B, with 90% of the patients in Group A achieving an articular step-off of less than 1 mm after reduction. Fracture healing relies on adequate blood supply and preservation of soft tissues (25, 26). The patella is covered by thin skin and minimal soft tissue, making it prone to delayed union or nonunion after fracture; thus, protection of its vascular supply and surrounding soft tissues is particularly critical during surgery. Previous studies have reported that the patellar blood supply primarily arises from the medial aspect and that the lateral parapatellar approach does not significantly compromise patellar vasculature while avoiding injury to the infrapatellar branch of the saphenous nerve (27, 28)—further highlighting its advantages. In general, complex comminuted patellar fractures are associated with severe prepatellar soft tissue injury. Adopting an anterior longitudinal incision in such cases may further damage soft tissues and the prepatellar vascular network. Additionally, longitudinal soft tissue dissection, which lacks traction from a soft tissue hinge, increases the separation of comminuted fragments, exacerbating the difficulty of reduction. In contrast, the lateral parapatellar approach allows for intact flap elevation, avoiding the aforementioned soft tissue and vascular injuries. Eversion of the patella facilitates reduction by visualizing the articular surface, indirectly preserving the periosteal blood supply to the superior patellar surface and promoting fracture healing, which is consistent with the statistical analysis of fracture healing times between the two groups. Although the lateral vascular supply is less critical than the medial supply for the patella, meticulous soft tissue handling during surgery remains essential to minimize lateral vascular injury, thereby maintaining patellar perfusion, accelerating fracture healing, and reducing patellofemoral complications (28).

Healing of comminuted patellar fractures is predicated on stability, and reliable internal fixation is critical to achieve this stability. Comminuted fractures require fixation with multiple Kirschner wires, and the lateral parapatellar approach allows direct visualization of the patellar articular surface, preventing accidental penetration of the joint cavity during wire insertion. This approach ensures that wires are placed close to the articular cartilage layer, enhancing fixation anchorage and stability and facilitating early mobilization without impeding joint functional exercises. Consistent with the follow-up results, Group A patients initiated knee functional exercises significantly earlier than Group B patients did, with superior joint function outcomes. The placement of Kirschner wires outside the joint cavity facilitates exposure of their tail ends, avoiding re-entry into the joint space during removal after fracture healing and simplifying extraction. Additionally, this approach reduces complications such as skin irritation caused by wire migration. Direct visualization of fracture fragments during reduction aids in precise fragment approximation and shortens the operative duration, as evidenced by the statistically significant shorter surgical time in Group A than in Group B.

Although there was no statistically significant difference in incision length between the two groups, the postoperative complication analysis revealed that Group B exhibited more severe hypertrophic scarring at the incision site, with a statistically significant difference (*P* < 0.05). This finding may be attributed to the location of the anterior incision, as the anterior longitudinal incision is subjected to greater tension during knee flexion and extension. Additionally, the higher VAS score in Group B on the third postoperative day was also associated with the increased tension at the anterior incision site.

There is no standardized criterion for selecting internal fixation materials in patellar fracture surgery; the choice of fixation materials should be individualized according to the specific fracture morphology. For complex comminuted patellar fractures, we adopted the “hedgehog technique” intraoperatively, supplemented by cerclage and fixation with Ethibond sutures. On the basis of precise anatomical reduction, this approach achieves rigid internal fixation, facilitating early knee flexion-extension exercises. In patients with complex comminuted patellar fractures, the bone fragments are too small to be fixed with cannulated screws, and plates also fail to provide effective fixation. Kirschner wires, which are available in various specifications, can meet the fixation requirements of small bone fragments, offering advantages of flexibility, convenience, and low cost. When combined with the wire tension band technique, they help create compression between bone fragments. With advancements in manufacturing processes, the strength of sutures has increased, and their antibacterial properties can also promote fracture healing (14, 29). Sutures serve as an excellent adjuncts and supplements to other implants; particularly for tiny bone fragments, the transosseous suture technique can achieve satisfactory reduction and maintain initial stability. In terms of fixation material selection for comminuted patellar fractures, the combination of multiple Kirschner wires with auxiliary suture fixation has shown favorable outcomes.

## 5 Conclusion

In the surgical management of complex comminuted patellar fractures, the lateral parapatellar approach, leveraging its unique anatomical advantages, minimizes iatrogenic injury to blood vessels, nerves, and the extensor mechanism while providing a clear surgical field for precise reduction of articular fragments. This approach yields significant therapeutic efficacy, obviates the need for patellectomy, and is worthy of clinical promotion and application. Despite the rapid evolution of orthopaedic techniques, the simple and cost-effective combination of Kirschner wire tension band fixation with Ethibond suture augmentation remains a robust and viable option, demonstrating enduring clinical value in the treatment of such fractures.

## 6 Limitation

This study has several limitations. First, it was a single-center, retrospective study with a small sample size. Second, the follow-up duration was short, resulting in a lack of data on the incidence of post-traumatic arthritis.

## Data Availability

The original contributions presented in the study are included in the article/Supplementary Material, further inquiries can be directed to the corresponding author.
